# Long-Term Management of Pulmonary Embolism: A Review of Consequences, Treatment, and Rehabilitation

**DOI:** 10.3390/jcm11195970

**Published:** 2022-10-10

**Authors:** Anette Arbjerg Højen, Peter Brønnum Nielsen, Thure Filskov Overvad, Ida Ehlers Albertsen, Frederikus A. Klok, Nanna Rolving, Mette Søgaard, Anne Gulbech Ording

**Affiliations:** 1 Unit for Thrombosis and Drug Research, Aalborg University Hospital, 9000 Aalborg, Denmark; 2 Department of Cardiology, Aalborg University Hospital, 9000 Aalborg, Denmark; 3 Aalborg Thrombosis Research Unit, Department of Clinical Medicine, Faculty of Medicine, Aalborg University, 9000 Aalborg, Denmark; 4 Department of Clinical Pharmacology, Aalborg University Hospital, 9000 Aalborg, Denmark; 5 Department of Medicine–Thrombosis and Hemostasis, Leiden University Medical Center, 2333 Leiden, The Netherlands; 6 Department of Physical and Occupational Therapy, Aarhus University Hospital, 8200 Aarhus, Denmark; 7 Department of Public Health, Aarhus University, 8200 Aarhus, Denmark

**Keywords:** pulmonary embolism, long-term management, cancer-associated thrombosis, anticoagulation, rehabilitation, bleeding, patient preferences, recurrence, review

## Abstract

The concept of pulmonary embolism is evolving. Recent and emerging evidence on the treatment of specific patient populations, its secondary prevention, long-term complications, and the unmet need for rehabilitation has the potential to change clinical practice for the benefit of the patients. This review discusses the recent evidence from clinical trials, observational studies, and guidelines focusing on anticoagulation treatment, rehabilitation, emotional stress, quality of life, and the associated outcomes for patients with pulmonary embolism. Guidelines suggest that the type and duration of treatment with anticoagulation should be based on prevalent risk factors. Recent studies demonstrate that an anticoagulant treatment that is longer than two years may be effective and safe for some patients. The evidence for extended treatment in cancer patients is limited. Careful consideration is particularly necessary for pulmonary embolisms in pregnancy, cancer, and at the end of life. The rehabilitation and prevention of unnecessary deconditioning, emotional distress, and a reduced quality of life is an important, but currently they are unmet priorities for many patients with a pulmonary embolism. Future research could demonstrate optimal anticoagulant therapy durations, follow-ups, and rehabilitation, and effective patient-centered decision making at the end of life. A patient preferences and shared decision making should be incorporated in their routine care when weighing the benefits and risks with primary treatment and secondary prevention.

## 1. Introduction

Venous thromboembolism, comprising deep vein thrombosis and pulmonary embolism, affects approximately 10 million people globally each year [1]. Pulmonary embolism-related death rates have declined, yet one in five patients die within one year after diagnosis [2,3]. Several subgroups of patients pose a particular challenge for thrombosis treatment, and the guidelines are often unspecific regarding its long-term management. Pulmonary embolism frequently recurs and it is associated with long-term disability, patient distress, and anticoagulant-related bleeding [1]. Overall, the concept of venous thromboembolism is undergoing a major transition in the scientific and clinical community, and it is increasingly considered a chronic illness [4]. In this narrative review, we discuss the current evidence on anticoagulation treatment, rehabilitation, emotional stress, quality of life, and the associated outcomes for patients with a pulmonary embolism.

## 2. Consequences of Pulmonary Embolism

The complications of a pulmonary embolism reduce the patient’s quality of life and are associated with substantial health care costs [5,6]. A pulmonary embolism may result in long-term negative consequences that can affect the patients’ ability to resume everyday life for months or years. The physical symptoms like dyspnoea, dizziness, chest pain, and discomfort during physical activity may continue and even become a chronic condition, although no residual thrombus is visible in the scans that are taken [7,8,9,10]. In addition to the physical symptoms, many patients report signs of psychological distress, e.g., anxiety and sleep problems, which all together, reduce the patients’ motivation for resuming a normal, physically active everyday life [9,11,12,13].

### 2.1. Physical Function

Despite the effective medical treatment of pulmonary embolism, many patients report negative long-term consequences. Persisting dyspnea, fatigue, reduced physical capacity and exercise intolerance, which is also known as post-pulmonary embolism syndrome, have been reported to affect up to half of the patients [7,10,14,15], while around 3% of percent of them develop the serious condition chronic thromboembolic pulmonary hypertension [6,14,16]. In a prospective cohort study with 100 patients with pulmonary embolism patients, 46.5% of the patients had exercise limitations which was assessed with a predicted peak oxygen uptake <80% and an increased pressure in the pulmonary circulation at a one year follow-up [10]. Not surprisingly, the reductions in their physical capacity were associated with a significantly worse health-related quality of life, dyspnea, and a reduced walking capacity (assessed with 6-min walk test). Deconditioning occurring after the pulmonary embolism event is the most likely explanation for the functional impairment that is reported in several studies, as no meaningful associations have been found between the heart or lung function and their physical capacity or the patient-reported symptoms [17,18].

Understanding the negative long-term effects of a pulmonary embolism on the physical function of the patient is complex, and the explanations of them are most likely multifactorial. Many patients with a pulmonary embolism also suffer from other health conditions that may contribute to these functional impairments, and psychological factors also play an important role [9,11,12,13]. Thus, resuming physical activity and sports often induces the fear of a new thrombus, and many patients are uncomfortable with initiating exercise after being discharged [9]. It has been demonstrated that functional impairment presents with similar frequency after a pulmonary embolism as it does among patients with coronary heart disease or stroke up to eight years after the pulmonary embolism [19], which underscores the need for rehabilitation.

### 2.2. Quality of Life and Emotional Distress

A large proportion of the patients with a pulmonary embolism experience a reduced quality of life, fatigue, and emotional distress [9,12,14,20,21,22,23,24,25]. The patients with elevated pressure in the pulmonary circulation and post-pulmonary embolism syndrome report that they have lower quality of life scores than population comparisons and patients with pulmonary embolism who have undergone full recovery [23,26,27,28]. Thus, a poorer quality of life has been demonstrated to depend greatly on functional impairment, and it correlates with a poor physical performance [23,26,27,28].

For the majority of these people, the quality of life improves in the year after the pulmonary embolism. In a 2017 prospective observational study of a Canadian cohort with 100 patients surviving a pulmonary embolism, being of the female sex, a higher body mass index (BMI), and exercise limitations at 1-month were the most notable clinical and physiological predicters of a reduced quality of life improvement over time [23]. A more recent German study confirmed that both the disease-specific as well as generic quality of life improved in the first year after a pulmonary embolism diagnosis, with female sex, a prior cardiopulmonary disease, and high BMI scores predicting a poorer quality of life and less improvement over time [29].

A recent study has shown that the patients report an inclination to seek support from mental health services [25]. However, despite the significant emotional distress and a poor quality of life being experienced by many patients following a pulmonary embolism, these long-term consequences are not systematically addressed in the current post-pulmonary embolism management guidelines. A recent paper of the European Society of Cardiology and European Respiratory Society, however, underlined the importance of measuring the long-term impact of pulmonary embolisms, and the need to use patient-reported outcomes for this purpose [30]. Moreover, an initiative to support the improvement of pulmonary embolism care has been undertaken by the International Consortium for Health Outcome Measurement with the development of a standard set of patient-centered venous thromboembolism outcomes [31,32].

## 3. Anticoagulation for Acute and Extended Treatment

Anticoagulation is the cornerstone of pulmonary embolism treatment. Anticoagulant therapy aims to reduce the mortality that is caused by pulmonary embolism and the morbidity of thrombus extension, recurrence, and post-thrombotic syndrome. The hemodynamically unstable patient with a pulmonary embolism also initially requires a reperfusion treatment, e.g., thrombolytic therapy, surgical embolectomy, or a percutaneous catheter-directed treatment [33]. For decades, the only options for the treatment and prevention of venous thromboembolism were a vitamin K antagonist (VKA, principally warfarin), unfractionated heparin, or low-molecular-weight heparin (LMWH). Although effective, these agents are also associated with major disadvantages, such as inter- and intra-individual pharmacokinetics and pharmacodynamics, or the need for subcutaneous and/or intravenous delivery. VKAs are sensitive to many drug–drug interactions as well as vitamin K intake and are thus influenced by dietary habits and fluctuations in, e.g., alcohol intake, but also long-term changes such as serum albumin levels due to protein binding. This is reflected in there being significant changes in the International Normalized Ratio (INR) values. The treatment with VKA typically requires regular visits to outpatient clinics or general practitioners for routine blood testing and potential dose adjustments. In a clinical setting, the management of warfarin is—at best—cumbersome.

These treatment-related issues led, more than a decade ago, to the development of a new class of drugs, the direct oral anticoagulants (DOACs). The DOACs have been studied and approved for the treatment of venous thromboembolism in various settings. The advantages of this class of drugs are that they can be given orally at a fixed dose (once or twice daily) and that there is no need for regular laboratory monitoring which is followed by dose adjustments. The DOACs act directly on the coagulation factors (thrombin or factor Xa) with a predictable pharmacokinetic profile.

Four randomized clinical trials investigated dabigatran [34], rivaroxaban [35,36], and apixaban [37] for extended treatment periods after a patient has been diagnosed with a pulmonary embolism, and these showed that the drugs effectively reduced the recurrence risk, but that this benefit was partially offset by an increased bleeding risk. Common for the four trials were a clinical equipoise between the continuation or no oral anticoagulant treatment and a requirement of undergoing up to 12 months of the initial treatment with either a DOAC or warfarin following venous thromboembolism. The patients were subsequently randomized to an active treatment or placebo (although two trials had an active treatment in the placebo arm) [34,36]. The summary of the findings from the anticoagulant extension trials are listed in Table 1. All of the active treatment arms displayed a reduced risk of venous thromboembolism recurrence apart from the RE-MEDY study in which 150 mg dabigatran that was administered twice daily vs. warfarin was associated with a higher recurrence risk [34].

The decision on the treatment duration beyond the conventional period of at least 3 months is based upon the estimated risk of recurrence when the anticoagulant treatment is stopped. In the PADIS-PE trial, the patients with deep vein thrombosis were randomized to have an additional 18 months warfarin treatment vs. a placebo after a fixed 6 months initial warfarin treatment following the pulmonary embolism, thereby demonstrating that there is a reduced risk of recurrent venous thrombosis and major bleeding [40]. The existing trials have only investigated the extended treatment with DOACs in venous thromboembolism populations also including deep vein thrombosis. Thus, no trial has specifically investigated the recurrence risks that are associated with an extended DOAC treatment among patients with a pulmonary embolism. The results from an observational study suggested that an extended anticoagulant treatment for pulmonary embolism over 2 ½ years was more effective and safer in comparison to having no extended therapy [41].

The decision to undergo an indefinite OAC treatment must be balanced against the risk of bleeding [42]. Importantly, the bleeding itself may not be directly caused by an anticoagulant drug, but the severity is likely to be intensified because of the effect that it has on the coagulation pathway. In the four randomized trials, the DOAC treatment was associated with an increased risk of major bleeding in comparison with placebo or aspirin groups which showed no increased risk of major bleeding, but this was not the case in the warfarin group. The populations that were enrolled in the trials were generally younger (avg. age 56 years) and likely had fewer comorbidities because of the trial exclusion criteria, and thus, they may not reflect a more general pulmonary embolism population. The trial results generally confer a positive net clinical benefit in favor of extended treatment, yet the question remains on how to select to patients who will have the greatest benefit from an extended treatment. Deciding among treatment options (aspirin, DOAC, or no treatment) requires an individual assessment to be made of the perceived bleeding and recurrence risks. The patients with a high risk of recurrence, or who would have more severe consequences in case of recurrence, are most likely to derive the highest benefit from a DOAC treatment.

Only a few studies have investigated the extended treatment regimen in venous thromboembolism [43,44]. In Denmark, less than 5% of the patients initiated an extended treatment with 10 mg rivaroxaban or 2.5 mg apixaban which was administered twice daily after an initial treatment period of at least 3 months between 2017 and 2018 [45]. Using US claims data, DeRemer et al. investigated the extended treatment with 2.5 mg vs. 5 mg apixaban among more than 6000 patients with venous thromboembolism who had completed 6 months of an initial treatment [46]. The authors concluded that 2.5 mg apixaban was associated with similar outcomes as those which were seen with the higher dose, but this study highlights limitations such as confounding by indication, given the nature of the observational study design. In a recent observational cohort study from France with 1199 patients with pulmonary embolisms that were diagnosed during 2012–2015 who had survived the initial six months after their diagnosis, 71.5% of them received an extended treatment for at least two years with DOAC or VKA following the pulmonary embolism. A comparison of the long-term risk of adverse events among patients with a pulmonary embolism receiving an extended treatment revealed that there was a 2.1% risk of all-cause death or recurrent venous thromboembolism among the patients receiving the extended treatment versus 7.7% risk of the same among the patients who were not receiving an extended treatment (*p* < 0.001) [41].

From the patient perspective, the qualitative studies on the patient’s preferences and experiences have shown that patients who were treated for venous thromboembolism had a greater concern for the recurrent events than for the potential harm of the treatment. Although the majority of the patients wished to avoid adverse events, only 21–25% were afraid of bleeding events [47,48,49]. The vast majority (87%) of the patients who had experienced a bleeding episode reported that they were “not afraid of” bleeding events [50].

## 4. Guidelines on Long-Term Management

Guidelines for the management of pulmonary embolisms have been published by leading international societies, including the American College of Chest Physicians [51], the American Society for Hematology [52], and the European Society of Cardiology [33], and specific guidelines exist for cancer-associated thrombosis [53,54,55,56,57]. Treatment in a cancer setting is discussed in a separate section that is below this one. In 2014, the extended treatment option for venous thromboembolism prophylaxis was included in the international European guidelines [58]. During long-term anticoagulation treatment, all guidelines recommend a periodical, routine clinical reassessment of the pulmonary embolism recurrence and bleeding risk, drug–drug interactions, renal function, and the patients’ adherence to the treatment considering the patients’ preferences and their clinical situation. However, there is no evidence supporting the avoidance of long-term anticoagulation treatments in patients with a high estimated bleeding risk based on the existing bleeding risk stratification tools [59]. Instead, this knowledge should be discussed with the patients and incorporated in a shared decision-making process about the continuation or discontinuation of anticoagulation treatments [30]. In regard to the acknowledgement of the unmet needs and serious negative consequences for pulmonary embolism survivors, the European Society of Cardiology 2019 guidelines on pulmonary embolism management further recommends an integrated model of patient care after an acute pulmonary embolism that involves hospital specialists, appropriately qualified nurses, and primary care physicians to ensure the optimal transition from hospital to community care [33].

The guideline also endorse the provision of rehabilitation for patients with persisting symptoms including exercise, behavioral education, and risk factor modification [33].

However, few studies have investigated the effect of such integrated care models, and the content and criteria for identifying patients that need such post-pulmonary embolism care and rehabilitation remain unclear.

## 5. Recommendations for Specific Populations

Evidence-based clinical practice guidelines recommend DOACs as they are preferred choice for most patients with non–cancer-related venous thromboembolism [33].

Of note, DOACs are not recommended in patients with a severe renal impairment, during pregnancy and lactation, and in patients with antiphospholipid antibody syndrome or mechanical heart valves [33,60].

### 5.1. Cancer

Patients with cancer pose a particular challenge with respect to venous thromboembolism, as reflected by the multiple thrombosis guidelines that are dedicated to this specific population [61,62]. The incidence of both venous thrombosis, bleeding, and recurrent venous thrombosis is high, thereby complicating the decisions on treatment and secondary prophylaxis [63,64,65]. Adding to this, the treatment decisions are often taken by the treating oncologist or hematologist without their consultancy with a thrombosis specialist [66]. The guidelines typically recommend a minimum of 6 months of an anticoagulation treatment after cancer-associated venous thromboembolism [61]. As the recommendations are usually provided collectively for deep vein thrombosis and pulmonary embolism, they are not specifically discussed for pulmonary embolism in this section. The guidelines provide roughly similar recommendations for the treatment duration, with some variation regarding the selection of candidates for an extended treatment.

The routine treatment and secondary prevention for cancer-associated venous thromboembolism has consisted of LMWH for decades [67,68]. The DOACs have recently been evaluated for this indication and found to be valuable oral alternatives for selected patients with cancer [69,70]. While LMWH is still the primary preferred anticoagulant drug class for patients with gastrointestinal (in particular upper gastrointestinal) and urogenital tract cancer, DOACs are recommended as first mode of treatment in most other patients with cancer-associated thrombosis. Yet, uncertainties remain regarding their treatment beyond the initial 6 month treatment period [71]. The guidelines opt for the continued an anticoagulant treatment, but the recommendations are only sparsely supported by randomized evidence, and they are generally without reference to dosage or specific decision tools to aid these challenging decisions [61,72].

#### Guidelines for Cancer Patients

The National Comprehensive Cancer Network (NCCN) and American Society of Clinical Oncology (ASCO) guidelines provide the most specific, but also inclusive, recommendations, advocating that all patients with active cancer or ongoing cancer therapy continue their treatment beyond the initial treatment period (NCCN: 3 months; ASCO: 6 months) [56,57]. The 2019 International Initiative on Thrombosis and Cancer (ITAC) guidelines recommend that a treatment beyond 6 months ‘should be based on individual assessment of benefit-risk ratio, tolerability, drug availability, patient preference, and cancer activity’ [73]. The 2019 European Society of Cardiology guidelines suggest that they should undergo a minimum of 6 months of this treatment, with the option of undergoing a continued treatment in case of them having active cancer, but also allow for the continued treatment for those with cancer in remission based on case-by-case evaluation [33].

The incidence of adverse events due to a continued treatment beyond an initial 6-month treatment period has been investigated in both observational and randomized settings, although randomized evidence remains scarce [74]. Observational data suggest that the incidence of recurrence is highest in the first 6 months after the initial event, with lower incidences occurring in the following 6 months [74]. However, all of the observational studies are, by nature, restricted to describing the clinical course of the extended treatment only for those patients for which the treating physician has deemed a continued treatment suitable [74]. Specifically, patients who are not continuing their treatment do so for a certain reason (clinically assessed to be low risk of venous thromboembolism or at high risk of bleeding, patient refusal, terminal illness, etc.) which renders the patients who are continuing their treatment to be unrepresentative of the population of interest.

The randomized trials on extended anticoagulation treatments in cancer patients are summarized in Table 2. The only randomized studies that tested treatment vs. no treatment beyond 6 months are the SELECT-D: 12 m study and the Cancer-DACUS trial [75,76]. Both of these demonstrated that there were lower incidences of venous thromboembolism recurrence, but a higher risk of bleeding with a continued treatment versus the placebo group. There are a limited number of randomized studies that are available which have been performed only in highly selected patients such as those with documented residual deep vein thrombosis after an initial treatment (Cancer-DACUS) or in patients who were included only at ‘the discretion of the physician’ (SELECT-D: 12 m) [75,76]. Thus, there are no definitive existing or ongoing studies—randomized nor observational—that directly support the current guideline recommendations of a continued treatment beyond 6 months versus no treatment. The EVE and API-CAT trials are two very similar trials that aimed to assess the effect on 2.5 mg apixaban which was administered twice daily versus 5 mg apixaban which was administered twice daily in patients with cancer who have completed 6–12 months of an initial anticoagulant treatment for venous thromboembolism (Table 2) [77,78]. Although the HOKUSAI-VTE and the ongoing EVE and API-CAT trials will shed light on the comparisons between various active treatment strategies, they do not include a placebo arm. As such, the scientific foundation for recommending an extended treatment versus a treatment cessation in the first place is puny, particularly considering the absolute number of patients that are affected by these guidelines on a global scale. Although it is likely that the extended treatment is the optimal approach for most patients with cancer, evidence supporting this claim is essentially absent. Also, there are no randomized trials, and there are currently very limited observational data providing insight into the use of anticoagulation beyond 12 months [79].

### 5.2. Managing Anticoagulation at the End of Life

Pulmonary embolism commonly occurs in patients receiving palliative end-of-life care. However, while the clinical guidelines provide defined thresholds for starting an anticoagulation treatment, there is no clear guidance for tailoring the length of the anticoagulation treatment near the end of life. Instead, clinicians are asked to engage in shared decision-making with the patients and their families [33,52,53,55,56,57,73,80]. Data about the potential benefits and harms are essential for this discussion, but little evidence is available. The randomized controlled trials of efficacy and safety of an anticoagulation treatment excluded patients with poor performance status or a life-expectancy below 3–6 months, thereby under-representing patients who are approaching the end of life, where the benefit–risk balance of anticoagulation therapy is likely to change substantially.

When extrapolating the evidence from the studies of healthier trial populations, clinicians need to consider the fundamental changes in the goals of care at the end of life, where the quality of life and symptom relief may be more important than the survival and risk of recurrent pulmonary embolism is. Due to the lack of evidence, the clinicians may tend to overestimate the benefits of anticoagulation treatments and be inclined to continue a potentially inappropriate treatment in the last phase of life [81]. Accordingly, studies have shown that up to 50% of patients with life-limiting diseases continue to use antithrombotic treatment in the last weeks of life [82,83,84]. This may unnecessarily result in bleeding complications, suboptimal quality of life, or death for patients with a life-limiting illness. A lack of evidence on the risks and benefits of (dis)continuing anticoagulation treatments in the terminal phase of life is one of the most important barriers to informed decision making and the widespread implementation of deprescribing of it, and it points to the need for high-quality data to address this.

## 6. Rehabilitation

As previously described, studies have demonstrated that many patients with pulmonary embolism experience functional impairment, even years after the pulmonary embolic event. This is in part due to a vicious circle where the symptoms of dyspnoea and fatigue which are combined with emotional distress lead to increased inactivity over time, thereby resulting in a reduced physical capacity and deconditioning, which in turn maintains the experience of exercise intolerance, dyspnoea, and fatigue. Thus, continued symptoms and reduced physical capacity is not necessarily caused by any disease-related factor per se, but often results from physical inactivity. These symptoms and functional limitations can therefore be improved with a rehabilitation program that includes exercise. Several studies have investigated the safety and effect of initiating physical exercise after a pulmonary embolism, varying in the study design and interventions that were used [85,86,87,88,89,90,91,92]. The interventions included cardio exercise (running, biking, and fast walking) and these were initiated within a few weeks to several months after the pulmonary embolism was diagnosed, and some included exercise intervals with increased intensity of the program (e.g., bouts of high intensity for 2–3 min several times during each training session).

In general, there were no adverse events which were related to the exercise interventions, e.g., recurrent pulmonary embolism (or deep venous thrombosis) or bleeding. In terms of its effect, physical fitness and mental well-being including less dyspnoea and exhaustion during activity, functional ability, energy to perform everyday activities, quality of life, and sleep quality improved. These positive effects can most likely be explained by several mechanisms, which are both physiological and psychological. As the physical and mental energy increases along with increased physical capacity and strength, many patients even experience a feeling of self-efficacy and empowerment in the management of their condition.

## 7. Conclusions

Patients with a pulmonary embolism are heterogeneous, and the recurrence risk varies considerably according to the patient’s characteristics [93]. A determination of the optimal duration of an anticoagulation treatment is recommended to be based on an assessment of a patient’s individual risk factors. For patients receiving an extended treatment, a yearly reassessment is recommended [33].

At yearly intervals, an estimated recurrence risk should be balanced against an estimated bleeding risk. However, this balance remains delicate, particularly for cancer patients and patients at the end of life where there is very limited evidence for prescribing an extended treatment. Both the bleeding and recurrence risk can be difficult to estimate; the risk cut-off values for extending/stopping treatment are uncertain and finally, patient profiles are dynamic with factors affecting bleeding and recurrence risks which change over time.

Several unanswered questions remain. First, the guidelines focus primarily on risk assessment, prophylaxis, diagnosis, and initial anticoagulant treatment, and no clear guidance is available on how to select patients who will have a net clinical benefit from receiving an extended treatment that is beyond three to six months, in particular for patients with cancer-associated thrombosis. The guidelines allow for an indefinite anticoagulant treatment, yet these recommendations are not strongly supported by high-quality studies and do not aid in decision making regarding the optimal dosage, duration, and discontinuation. Finally, there is a need for more information regarding the effect of integrated care models and the content and criteria for identifying patients that are in need of post-pulmonary embolism care and rehabilitation.

## Figures and Tables

**Table 1:** Characteristics of studies on extended anticoagulation after incident VTE (adapted from Albertsen et al.).

Intervention *	Study, Year	Comparison	No. Patients Enrolled	Patients with Index PE	Treatment Duration	Recurrence Proportion (%) in Intervention * vs. Comparison Group	Recurrence risk: HR; 95% CI	Major or CRNM Bleeding in Intervention * Group: HR; 95% CI
**Dabigatran**	RE-SONATE, 2013 [34]	Placebo vs. dabigatran 150 mg BID	1343	33%	6–18 months	0.4% vs. 5.6%	0.08; 0.02–0.25	2.92; 1.52–5.60
	RE-MEDY, 2013 [34]	Warfarin vs. dabigatran 150 mg BID	2856	35%	18–36 months	1.8% vs. 1.3%	1.44; 0.78–2.64	0.54; 0.41–0.71.
**Rivaroxaban**	EINSTEIN Extension, 2010 [35]	Placebo vs. rivaroxaban 20 mg OD	1196	38%	6–12 months	1.3% vs. 7.1%	0.18; 0.09–0.39	5.19; 2.3–11.7.
	EINSTEIN Choice, 2017 [36]	Aspirin 100 mg OD vs. rivaroxaban 20 mg OD rivaroxaban 10 mg OD	3365	49%	12 months	Riva 20 mg: 1.5% Riva 10 mg: 1.2% vs. Aspirin: 4.4%.	Riva 20 mg vs. aspirin: 0.34; 0.20–0.59. Riva 10 mg vs. aspirin: 0.26; 0.14–0.47.	1.59; 0.94–2.69. 1.16; 0.67–2.03.
**Apixaban**	AMPLIFY Extension, 2013 [37]	Placebo vs. apixaban 5 mg BID vs. apixaban 2.5 mg BID	2486	35%	12 months	Apixaban 5 mg: 1.7% Apixaban 2.5 mg: 1.7% vs. Placebo: 8.8%	Apixaban 5 mg vs. placebo: 0.36; 0.25–0.53. Apixaban 2.5 mg vs. placebo: 0.33; 0.22–0.48.	1.62; 0.96–2.73. 1.20; 0.69–2.10.
**Aspirin**	WARFASA, 2012 [38]	Placebo vs. aspirin 100 mg OD	402	40%	≥24 months	6.6% vs. 11.2%	0.58; 0.36–0.93.	0.98; 0.24–3.96.
	ASPIRE, 2012 [39]	Placebo vs. aspirin 100 mg OD	822	30%	2–4 years	4.8% vs. 6.5%	0.74; 0.52–1.05.	1.1% per year with aspirin (vs. 0.6%).

BID = twice a day; CI = confidence interval; CRNM = clinically relevant non-major; HR = hazard ratio; OD = once a day; PE = pulmonary embolism; Riva = rivaroxaban; VTE = venous thromboembolism. * ‘Intervention’ denotes the anticoagulant drug tested in the table.

**Table 2:** Overview of randomized trials investigating the effectiveness and safety of anticoagulation beyond 6 months treatment in patients with cancer and venous thromboembolism.

Trial Name	Study Population	Treatment Comparison	Outcome			
			Recurrent Venous Thrombo-Embolism	Major Bleeding	Clinically Relevant Non-Major Bleeding	Other
*Completed trials*						
SELECT-D: 12 m	Ninety-two patients with cancer and residual deep vein thrombosis or index pulmonary embolism having completed 6 months of rivaroxaban or dalteparin *	Six months rivaroxaban 20 mg − 1 vs. placebo	4% vs. 14%, Hazard ratio 0.32 (0.06–1.58)	5% vs. 0%	4% vs. 0%	
Hokusai VTE Cancer–post hoc analysis	Five hundred and sixty-seven patients with cancer having completed 6 months treatment with either edoxaban or dalteparin **	Up to 6 months edoxaban 60 mg − 1 versus dalteparin 150 IU/kg ***	1.4% vs. 2.9%, Hazard ratio 0.48 (0.14–1.63)	2.4% vs. 1.1%, Hazard ratio 2.23 (0.59–8.46)	4.8% vs. 4.8%, Hazard ratio 1.02 (0.48–2.16)	Recurrent VTE or major bleeding: 3.7% vs. 4.0%, Hazard ratio 0.96 (0.42–2.22)
Cancer-DACUS ****	Two hundred and forty-two patients with cancer and residual vein thrombosis having completed 6-month treatment for deep vein thrombosis	Six months of nadroparin-75% of full weight-adjusted dosage-vs. no treatment	4 vs. 18 events	4 vs. 1 event	Not reported	Not reported
*Ongoing trials*						
EVE, NCT03080883	Three hundred and seventy patients who have completed 6–12 months of anticoagulation after cancer-associated venous thromboembolism	Twelve months of apixaban 2.5 mg bid vs. 5 mg bid	Yes	Yes–combined		Arterial thrombosis
API-CAT, NCT03692065	One thousand seven hundred and twenty-two patients with cancer who have completed 6 months of anticoagulation for proximal deep-vein thrombosis and/or pulmonary embolism	Twelve months of apixaban 2.5 mg bid vs. 5 mg bid	Yes	Yes		Recurrent symptomatic VTE VTE-related death All-cause death Major bleeding

* One hundred and thirty-six patients were eligible for randomization, but 44 patients declined to participate or were advised not to by their clinicians, e.g., due to bleeding risk assessment. ** The decision on treatment continuation beyond 6 months, including choice of anticoagulant, was left to the discretion of the treating physician, and thus not based on randomization. *** The initial randomization dosage was edoxaban 60 mg daily preceded by 5 days of low-molecular-weight heparin (with dose reductions to edoxaban 30 mg daily in patients with low body weight, impaired renal function, or concomitant strong P-gp inhibitors) versus dalteparin 200 IU/kg for 30 days followed by 150 IU/kg thereafter. **** Main results originally reported after 12-month follow-up, but the active treatment period was only 6 months. This table presents only data during the active treatment period.

## Data Availability

Not applicable.
